# Retraction: Transgenic Biofortification of the Starchy Staple Cassava (*Manihot esculenta*) Generates a Novel Sink for Protein

**DOI:** 10.1371/annotation/9081d3ad-e74f-47f1-a722-cf02048a14c5

**Published:** 2012-09-07

**Authors:** Mohammad Abhary, Dimuth Siritunga, Gene Stevens, Nigel J. Taylor, Claude M. Fauquet

Following the publication of this article, the authors have been unable to confirm the presence of the zeolin gene within the transgenic cassava plants in several subsequent studies. This raises concerns about the validity of the results reported in the article.

Additionally, the Committee on Research Integrity at Donald Danforth Plant Science Center has carried out an institutional investigation which revealed that significant amounts of data and supporting documentation that were claimed to be produced by the first author could not be found. Given that the validity of the results could not be verified, and in line with the recommendation issued by the corresponding author's institution, the authors retract the article.

The authors apologize to the readers.

